# Hepatic Decompensation Associated With COVID-19 in a Patient With Alcoholic Liver Cirrhosis: A Case Report

**DOI:** 10.7759/cureus.86615

**Published:** 2025-06-23

**Authors:** Martina Gengo

**Affiliations:** 1 Emergency Medicine, Institute for Emergency Medical Aid of Sarajevo Canton, Sarajevo, BIH

**Keywords:** alcoholic liver cirrhosis, covid-19, esophageal variceal bleeding, hepatic decompensation, hepatorenal syndrome

## Abstract

Coronavirus disease 2019 (COVID-19) presents a spectrum of severity, ranging from asymptomatic infection to life-threatening respiratory failure. Patients with comorbidities, such as alcoholic liver cirrhosis, are at increased risk for adverse outcomes, as COVID-19 may precipitate hepatic decompensation. We report the case of a 60-year-old man with a history of alcoholic liver cirrhosis who was admitted with fever, cough, diarrhea, and fatigue. COVID-19 was confirmed via polymerase chain reaction (PCR) testing. He was diagnosed with bilateral pneumonia and had elevated liver enzymes. Treatment included azithromycin, doxycycline, enoxaparin, and dexamethasone. The patient showed clinical improvement following 10 days of therapy and was subsequently discharged. Two months later, he developed gastrointestinal bleeding due to ruptured esophageal varices. Over the following months, his condition worsened progressively, marked by severe malnutrition, recurrent ascites, and the development of hepatorenal syndrome. Despite ongoing care, he died one year after the initial COVID-19 diagnosis. This case highlights alcoholic liver cirrhosis as an independent risk factor for COVID-19-related complications and mortality, underscoring the need for targeted acute management and long-term follow-up in this vulnerable population, an essential consideration for future infectious disease outbreaks.

## Introduction

Coronavirus disease 2019 (COVID-19) is a respiratory illness caused by the severe acute respiratory syndrome coronavirus 2 (SARS-CoV-2), first identified in Wuhan, China, in December 2019 [1]. Since then, the virus has rapidly spread across the globe, resulting in hundreds of millions of reported cases and over seven million deaths globally, according to the World Health Organization [2].

The clinical presentation of COVID-19 varies widely, ranging from asymptomatic infection to life-threatening respiratory failure. Common symptoms include fever, cough, fatigue, diarrhea, and loss of taste or smell [3]. While many cases are mild, others can progress to severe viral pneumonia, acute respiratory distress syndrome (ARDS), and death [4].

Patients with pre-existing comorbidities such as hypertension, diabetes, cardiovascular disease, and chronic respiratory conditions are known to have a higher risk of severe disease and mortality from COVID-19 [5]. Similarly, individuals with chronic liver disease are reported to have poorer outcomes. One study found that patients with cirrhosis had a 30-day mortality rate of 17.1%, were 4.1 times more likely to require mechanical ventilation, and 3.5 times more likely to die, while another retrospective study reported a 30-day mortality rate of 34% among individuals with cirrhosis infected with SARS-CoV-2 [6,7]. Complications such as ascites, hepatic encephalopathy, gastrointestinal bleeding, secondary infections, and liver failure may be exacerbated by SARS-CoV-2 infection, but further investigation is required [8].

Some studies have observed abnormal liver function tests in patients without prior liver disease, suggesting that SARS-CoV-2 may directly impair hepatic function. Furthermore, alcoholic liver disease has been independently associated with a 1.8-fold increased risk of COVID-19-related mortality [7,8]. Patients with alcoholic liver disease and COVID-19 also tend to experience more severe liver injury, with a significantly higher proportion having cirrhosis compared to patients with non-alcoholic fatty liver disease [7]. Additionally, studies have demonstrated a strong association between alcoholic liver damage and the severity of COVID-19, with odds ratios of 7.05 for alcoholic liver damage and 7.00 for alcoholic liver cirrhosis [7]. A case report further illustrates the poor outcomes in this population, including rapid progression to multi-organ failure and death following nosocomial COVID-19 infection during treatment for alcoholic liver disease [7].

This case report aims to present the clinical course of a patient with pre-existing alcoholic liver cirrhosis who contracted COVID-19 and subsequently developed progressive complications, highlighting the need for increased vigilance and comprehensive management in this high-risk population.

## Case presentation

A 60-year-old man with a known history of alcoholic liver cirrhosis presented to his general practitioner in November 2020 with fever (up to 39.5°C), dry cough, diarrhea, fatigue, and loss of appetite. A nasopharyngeal swab tested positive for SARS-CoV-2 by polymerase chain reaction (PCR), and he was admitted to the hospital shortly thereafter due to bilateral pneumonia and elevated liver enzyme levels. Initial laboratory workup revealed anemia, elevated inflammatory markers, and abnormal liver function tests. Key results are summarized in Table 1.

**Table 1:** Key blood test results at the time of COVID-19 diagnosis. COVID-19: coronavirus disease 2019; LDH: lactate dehydrogenase; CRP: C-reactive protein; AST: aspartate aminotransferase; ALT: alanine aminotransferase.

Parameter	Value	Reference range
Erythrocytes	3.84 x 10^12^/L	4.34-5.72 x 10^12^/L
Lymphocytes	4.4%	20-46%
Neutrophils	92.8%	45-72%
Thrombocytes	298 x 10^9^/L	158-424 x 10^9^/L
LDH	665 U/L	0-247 U/L
CRP	51.8 mg/L	0-5 mg/L
AST	202 U/L	8-38 U/L
ALT	57 U/L	10-48 U/L

The patient was treated with antipyretics and received antimicrobial and anti-inflammatory therapy as follows: azithromycin 500 mg orally once daily for three days, and doxycycline 100 mg orally twice daily for seven days. Dexamethasone was administered intravenously at a dose of 6 mg once daily for 10 days during hospitalization. Anticoagulation therapy was initiated with enoxaparin 100 mg subcutaneously twice daily for 10 days.

After completing the treatment course, the patient showed clinical improvement, with normalization of laboratory parameters (CRP: 20.1 mg/L; LDH: 371 U/L; AST: 38 U/L; ALT: 24 U/L), and was subsequently discharged. Upon discharge, enoxaparin was continued at 80 mg subcutaneously once daily for seven days, followed by aspirin 100 mg orally once daily for thromboprophylaxis, which was recommended for three months. The patient was also advised to avoid strenuous physical activity, maintain adequate hydration, and strictly abstain from alcohol during the recovery period.

Prior to the COVID-19 infection, the patient’s liver disease was clinically stable, with compensated cirrhosis. He had grade I esophageal varices managed with beta-blockers, as well as mild pitting edema and small-volume ascites, both effectively controlled with spironolactone and furosemide. He had no other known comorbidities. Key blood test results from one year prior to the COVID-19 infection are summarized in Table 2.

**Table 2:** Key blood test results one year prior to the COVID-19 diagnosis. COVID-19: coronavirus disease 2019; LDH: lactate dehydrogenase; CRP: C-reactive protein; AST: aspartate aminotransferase; ALT: alanine aminotransferase.

Parameter	Value	Reference range
Erythrocytes	4.47 x 10^12^/L	4.34-5.72 x 10^12^/L
Lymphocytes	22%	20-46%
Neutrophils	63.9%	45-72%
Thrombocytes	169 x 10^9^/L	158-424 x 10^9^/L
LDH	196 U/L	0-247 U/L
CRP	5 mg/L	0-5 mg/L
AST	36 U/L	8-38 U/L
ALT	29 U/L	10-48 U/L
Creatinine	85 µmol/L	45-90 µmol/L
Urea	7.0 mmol/L	2.0-7.8 mmol/L

However, two months after hospital discharge, the patient presented with hematemesis and melena and was diagnosed with hemorrhagic shock secondary to ruptured esophageal varices. Emergency endoscopic intervention successfully achieved hemostasis, and the patient was stabilized and discharged.

Over the following months, his condition progressively deteriorated. He developed severe malnutrition, fatigue, cognitive changes, and recurrent massive ascites requiring regular paracentesis. Laboratory tests revealed anemia (erythrocytes: 3.64 × 10¹²/L; reference range: 4.34-5.72 × 10¹²/L), elevated LDH (317 U/L; reference range: 0-247 U/L), AST (47 U/L; reference range: 8-38 U/L), and GGT (95 U/L; reference range: 11-55 U/L), along with low albumin (33.5 g/L; reference range: 35-52 g/L) and normal ALT (16 U/L; reference range: 10-48 U/L).

The patient also reported decreased urination. Laboratory tests showed markedly elevated creatinine (1086 µmol/L; reference range: 45-90 µmol/L) and urea (48.0 mmol/L; reference range: 2.0-7.8 mmol/L). In September 2021, he was hospitalized and diagnosed with hepatorenal syndrome, necessitating the initiation of hemodialysis. Despite ongoing supportive care, the patient ultimately succumbed to multi-organ complications 25 days later, approximately one year after his initial COVID-19 diagnosis.

## Discussion

COVID-19 has been shown to affect individuals with pre-existing comorbidities more severely, placing them at a greater risk for adverse clinical outcomes. Among these, patients with liver cirrhosis, particularly those with alcohol-related cirrhosis, are more susceptible to hospitalization, hepatic decompensation, and increased mortality following SARS-CoV-2 infection.

In a study by Singh and Khan, which included 2,780 patients with COVID-19, 9% had pre-existing liver disease. These patients were significantly more likely to require hospitalization and had increased mortality, especially those with underlying cirrhosis [9]. Marjot et al. further identified alcohol-related liver disease as an independent predictor of poor outcomes, noting a 1.8-fold increase in mortality in this subgroup [8]. In our case, the patient had compensated alcoholic liver cirrhosis and developed bilateral pneumonia as a complication of COVID-19, requiring inpatient management and respiratory support. He was discharged after clinical improvement, but his condition worsened in the following months.

Abnormal liver biochemistry is frequently observed in patients with COVID-19, with reports indicating that 15-65% of patients have elevated liver enzymes [10]. In our case, the patient’s transaminases were markedly elevated during his acute infection (AST 202 U/L and ALT 57 U/L), supporting a potential link between SARS-CoV-2 and hepatic injury. Singh and Khan similarly reported elevated liver enzymes in patients with COVID-19, suggesting the possibility of direct viral-induced liver injury [9].

This effect of COVID-19 on patients with pre-existing liver disease can be explained with findings that SARS-CoV-2 binds to the angiotensin-converting enzyme 2 (ACE2) receptors, which are expressed in hepatocytes and cholangiocytes. When the virus binds to the ACE2 receptor, it gains access to cells and starts to dysregulate liver function [9]. However, the causative relationship remains debated. Feng et al. proposed that liver injury in COVID-19 may be multifactorial, resulting from systemic inflammation, hypoxia-induced damage, drug toxicity, or multiorgan failure [11]. Additionally, in a study on hepatic and gastrointestinal involvement in COVID-19, Musa emphasized the importance of considering drug-induced liver injury during the treatment of COVID-19, particularly with the use of antipyretics, antibiotics (e.g., macrolides, quinolones), and corticosteroids [12]. In our case, the patient received similar medications, including antipyretics, azithromycin (a macrolide), doxycycline (a tetracycline-class antibiotic), and dexamethasone (a corticosteroid), which may have contributed to hepatic dysfunction and subsequent cirrhosis-related complications. Furthermore, Wu et al., in their review, highlighted that antibiotics, particularly tetracyclines and macrolides such as azithromycin, may exacerbate liver damage [13]. These observations underscore the need for careful therapeutic decision-making in patients with pre-existing liver disease, as standard COVID-19 treatments may pose additional hepatic risks.

Regarding liver cirrhosis complications, in a study conducted by Bajaj et al., readmissions of patients diagnosed with both liver cirrhosis and COVID-19 were mostly due to liver unrelated reasons; however, there was one liver-related case for readmission, which was due to hepatic encephalopathy and hepatocellular carcinoma [14]. In our case, the patient was readmitted to the hospital due to ruptured esophageal varices.

In a study conducted by Marjot et al., it is noted that SARS-CoV-2 infection in patients with cirrhosis precipitates liver dysfunction, resulting in acute hepatic decompensation in 179 patients (46%) [8]. In their study, the decompensation events included: new or worsening ascites (28%), hepatic encephalopathy (27%), spontaneous bacterial peritonitis (3%), and variceal hemorrhage (3%) [8]. It is known that variceal hemorrhage is a life-threatening complication in patients with cirrhosis, and it happens in 40% of patients with compensated cirrhosis and 70% of patients with decompensated cirrhosis [15]. While there is limited evidence that SARS-CoV-2 can directly affect the gastrointestinal tract and cause variceal bleeding, there are studies that suggest that COVID-19 can precipitate hepatic decompensation, which was also observed in our case [8].

Following COVID-19, the patient’s clinical status continued to deteriorate. He experienced cachexia, recurrent ascites requiring paracentesis, and neuropsychiatric symptoms suggestive of hepatic encephalopathy. This progressive health decline is consistent with findings by Iavarone et al., who reported that COVID-19 can cause rapid clinical deterioration even in previously stable patients with cirrhosis [16]. In their study, out of 25 patients with Child-Pugh score A, more than a third of them had rapid liver function deterioration, so their Child-Pugh score progressed to class B/C; deterioration was even worse in patients with decompensated disease before infection with SARS-CoV-2 [16]. These findings are in accordance with our case, where the patient also experienced faster liver function deterioration. The authors also concluded that the 30-day mortality rate was higher in patients who had worse liver function [16].

Our patient developed hepatorenal syndrome, a serious complication of end-stage liver disease. Despite hemodialysis, he succumbed 25 days later, approximately one year after his initial COVID-19 diagnosis. The development of hepatorenal syndrome in our patient, following COVID-19 infection and pre-existing alcoholic liver cirrhosis, reflects a severe trajectory consistent with what has been reported in the literature. Hepatorenal syndrome typically emerges in advanced liver disease and is precipitated by factors such as gastrointestinal bleeding and large-volume paracentesis, both of which were present in this case [17]. As highlighted in recent studies, COVID-19 may exacerbate liver dysfunction and increase the risk of hepatorenal syndrome, particularly in patients with chronic liver disease, indicating a poor prognosis and high likelihood of multi-organ failure [13].

Long-term outcomes and post-discharge mortality in patients with cirrhosis following COVID-19 remain mostly unknown. Navvas et al., in their study about COVID-19 post-discharge mortality rate in a London district general hospital, concluded that age, male gender, PCR-confirmed COVID-19, and multiple comorbidities were all associated with higher in-hospital and post-discharge mortality [18]. However, comprehensive data on the post-hospitalization course of patients with cirrhosis remain limited, making it challenging to draw comparisons or perform prognostic assessments in our case. Nonetheless, numerous studies indicate that COVID-19 can precipitate hepatic decompensation, and that alcohol-related liver cirrhosis is an independent risk factor for increased COVID-19-related mortality.

## Conclusions

Patients with comorbidities tend to have poorer outcomes when infected with SARS-CoV-2, and alcohol-related liver cirrhosis is recognized as an independent risk factor for increased COVID-19 mortality. We presented a case of a patient who experienced rapid hepatic decompensation following COVID-19, which supports existing literature linking SARS-CoV-2 infection with liver disease progression. Further studies are necessary to clarify the mechanisms and extent of COVID-19’s impact on hepatic function. We hope this case contributes to the growing body of evidence and highlights the challenges faced by patients with pre-existing liver disease.

Managing COVID-19 in patients with cirrhosis demands a heightened level of clinical care. Treatment strategies must address both the viral infection and the underlying liver condition. Continuous monitoring is essential during both the acute and recovery phases, as these patients are at a higher risk of complications and mortality. In our case, the patient had pre-existing poor liver function due to cirrhosis, underwent intensive therapy (antibiotics and corticosteroids), and experienced multiple health complications in the months following COVID-19, ultimately resulting in death. This underscores the importance of structured post-discharge care. A multidisciplinary approach, incorporating rehabilitation, routine follow-ups, and optimized treatment, can support early detection and management of complications, potentially improving outcomes for this vulnerable population.
